# The regional cancer spectrum in Uganda: a population-based cancer survey by sub-regions (2017–2020)

**DOI:** 10.3332/ecancer.2024.1782

**Published:** 2024-09-30

**Authors:** Francis Okongo, Catherine Amuge, Alfred Jatho, Nixon Niyonzima, David Martin Ogwang, Jackson Orem

**Affiliations:** 1 Uganda Cancer Institute, Kampala 10216, Uganda; 2 Gulu Cancer Registry, St. Mary’s Hospital Lacor, Uganda Cancer Institute, Gulu 70515, Uganda

**Keywords:** cancer, spectrum, Uganda, sub-regions, population based, survey, 2017–2020

## Abstract

**Background:**

Accurate estimation of the burden of cancer in developing countries is a major public health concern for cancer prevention and control because of the limited coverage of population-based cancer registries (PBCRs). The cancer registration coverage status of Uganda was 11.90% and was not uniformly distributed in all regions of Uganda. This population-based survey was conducted to assess the burden of cancer in all the sub-regions of Uganda by site, sex and age group to accurately determine the cancer profile of Uganda by sub-region for a tailored intervention to mitigate cancer risk factors and burden.

**Methods:**

This study used existing administrative units of Uganda from which 55 districts emerged, forming 10 sub-regions as satellite population-based cancer registry study sites. Data on newly diagnosed cancer cases were retrospectively collected for the period 2017–2020 using a cancer notification form, entered into CanReg5 Software, exported to spreadsheets and univariate analysis was performed to determine the cancer spectrum, their proportions and crude rates by site, sex, age group and geographical location.

**Results:**

A total of 25,576 cancer cases were registered, up to 14,322 (56%) were in females and, male cancers were 11,254 (44%). The top five female cancers in all the sub-regions included cervical cancer (43%, *n* = 6,190), breast (22%, *n* = 3,200), esophagus (5.6%, *n* = 800), ovary (5.2%, *n* = 746), Kaposi Sarcoma (KS) (4.7%, *n* = 666) and other less common cancers (18.5%, *n* = 2,720). In males, the top five cancers included prostate cancer 25.1 % (*n* = 2,820), esophagus 15.1% (*n* = 1,704), KS 12.4% (*n* = 1,395), liver 8.8% (*n* = 989) and stomach 4.8% (*n* = 539), with other less common male cancers accounting for 33.8% (*n* = 3,807).

In all the sub-regions of Uganda, cancers of the esophagus, liver and KS are common in both males and females, but the number of males with these cancers is twice that of their female counterparts. In Rwenzori, Kigezi and Bugishu sub-Regions, there seems to be an increased risk of developing other skin cancers in females, while stomach cancers have been reported in both males and females. Most of the other sub-regions register emerging cases of only ovarian cancer in females. In children, the top three cancers included lymphoma, 33.9% (*n* = 653); soft tissue sarcomas, 20.8% (*n* = 400); malignant bone tumors, 15.8% (*n* = 305); myeloid-type leukemia, 13.8% (*n* = 265); and the other less common childhood cancers combined, 15.7% (*n* = 303). The proportion of childhood cancers is higher in the male child compared to the female at a ratio of 1.3:1.

**Conclusions:**

The sub-regional cancer spectrum in Uganda ranges from cervical cancer to breast, esophageal, ovarian and KS in females. Male cancers include prostate, esophageal, KS, liver and stomach cancers. Although the cancer profile is similar in most sub-regions of Uganda, except Ankole subregions with mountainous topography (Rwenzori, Kigezi, Bugisu), there has been significant variation in cancer profile, especially for males, where Non-Hodgkins Lymphomas is one of the cancers reported for Uganda by PBCRs in Gulu, and Kampala has been replaced by stomach cancers as one of the common male cancers in the sub-regions. These findings emphasize the need for the establishment and support of additional regional PBCRs and periodic population-based cancer surveys to accurately determine the burden of cancer, inform the establishment of regional cancer centers and guide national and sub-national cancer control programs in Uganda. Cancer surveillance systems using PBCRs should be part of the national cancer control program. Periodic population-based cancer surveys should also be conducted as part of Uganda’s demographic and health surveys in areas without PBCRs to inform the country comprehensively and accurately on the cancer burden to design robust cancer mitigation measures.

## Introduction

Non-communicable diseases (NCDs) are a major cause of morbidity and mortality worldwide. Over 25 million people die from NCD-related causes, with cancers accounting for approximately one-third, 9.9 million deaths annually and over 19.3 M new cancer cases are registered annually [1, 2]. In Uganda, NCDs account for 33% of mortalities every year and cancers contribute about one-third, 9% of these deaths [3]. Cancer-related mortalities and survival at Uganda Cancer Institutes are 80% and 20%, respectively [4].

The most common types of cancers in Uganda are mainly cancers attributable to infections, such as those caused by human papilloma virus, Epstein Barr virus and human herpes virus-8. Lifestyle factors, such as harmful alcohol intake, sedentary lifestyle, smoking and unhealthy diet, have also been linked to these common cancers in Uganda. According to the 2014, NCD risk factors survey, over 9.6% (*M* = 16.8%, *F* = 2.8%) of Ugandans smoke tobacco, 28.5% (*M* = 40.1%, *F* = 17.9%) consumed alcohol, while of these alcohol drinkers, 16.7% (*M* = 26.2%, *F* = 7.9%) were heavy consumers of alcohol or consumed more drinks on any occasion in the past 30 days [5]. These risk factors vary from one person or community to another. In the 2019/2020 household survey in Uganda, the findings implicated Karamoja Sub-regions for high consumption of harmful alcohol compared to Busoga and other sub-regions [6].

**Figure 1.**
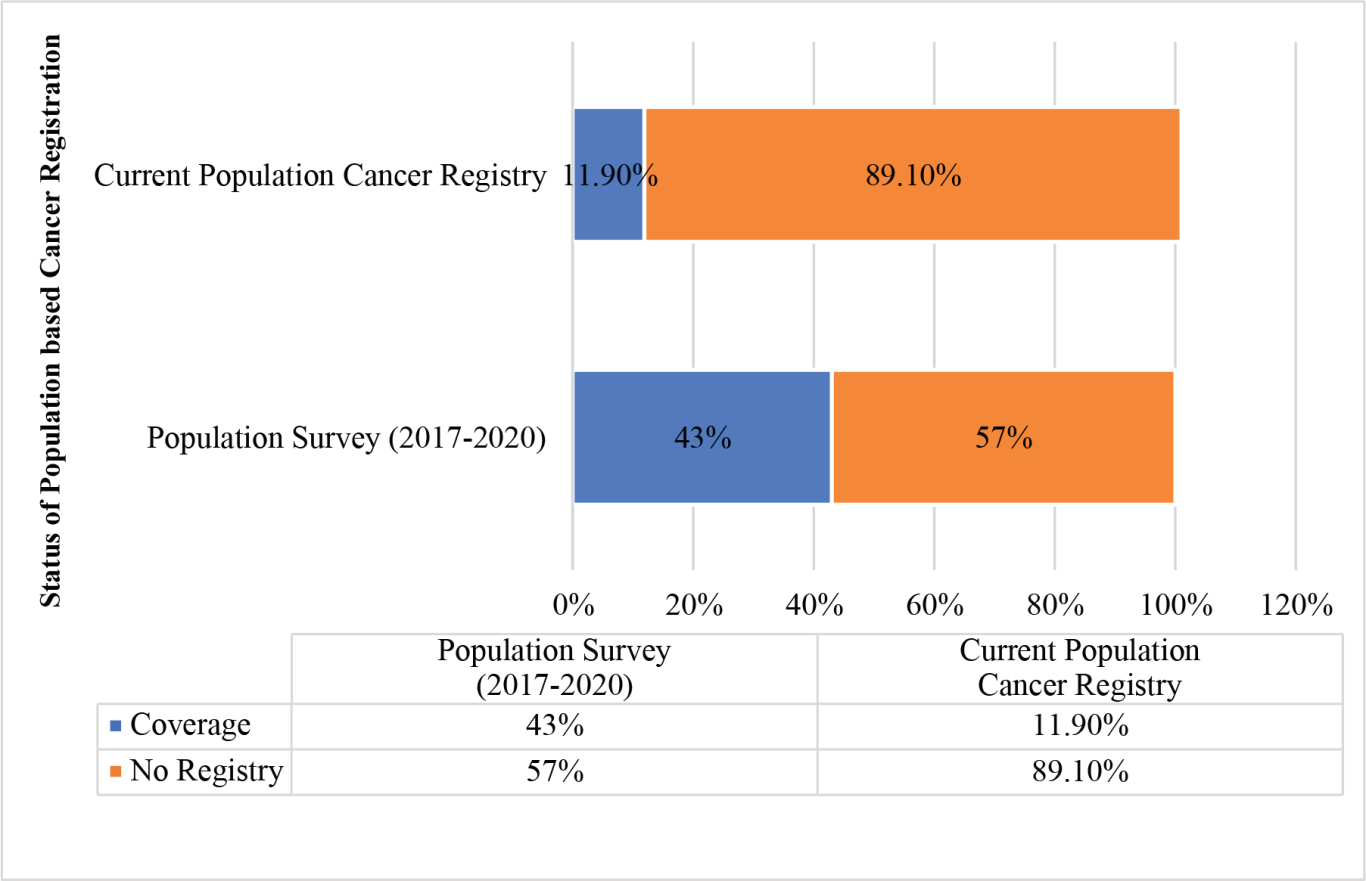
Coverage of the survey (2017–2020) and current population-based registry catchment.

The Uganda statistics on cancers in 2020 indicate that over 34,008 new cancers and 22,992 deaths were estimated using the Kampala and Gulu population-based cancer registries (PBCRs) [7]. The two population-based registry cancer datasets represent approximately 5 million people (11.9%) of the 42 million Ugandans [8]. The magnitude of cancer burden and profile by sub-region of Uganda is, therefore, not precisely known due to unequal distribution and low coverage of PBCRs in Uganda (Figure 1).

In many low- and middle-income countries, disease surveillance priority has been placed on communicable disease surveillance. It is paramount to understand and emphasize the need for NCD surveillance, particularly cancer surveillance [9]. Given the limited resources to establish PBCRs in all the sub-regions at the same time, there is a need to estimate the spectrum of cancers reported across the country to guide the prioritization of new PBCRs and to provide data that augment the available data provided by the existing PBCR to guide the national cancer control program. A snapshot of this cancer spectrum in the country is vital to guide efforts and investment to establish and maintain PBCR to ensure accurate documentation of the impact of national cancer control interventions and to select priorities for cancer control [10].

Based on this background, this population-based survey of common cancers by sub-region was conducted to assess the cancer spectrum in the different sub-regions of Uganda, looking at new cancer cases diagnosed between 2017 and 2020. The aim was to realistically understand the burden of cancer in the country at any one time and to provide a basis for setting additional PBCRs and regional cancer centers in the countryside.

## Methods

### Cancer registration methods and materials used

Population-based retrospective data collection was performed on cancer information from all public and private health facilities at the level of health center four, general, regional and national hospitals, cancer hospitals and pathology laboratories in 55 districts forming ten sub-regions of Uganda for the period 2017–2020. Cancer data were also extracted from private laboratories, palliative and hospice care centers, the mortuary records, among other source health facilities. Using the Cancer Notification Form (Appendix 1), information collected included cancer incidence dates, age, sex, usual address, tribe, site of cancer, cancer type, basis of diagnosis and status at last contact. Data quality checks were performed on-site for validity, completeness and consistency as per the World Health Organization International Classification of Diseases for Oncology Version 3 (ICD-O3)/International Classification of Diseases Version 10 (ICD-10) cancer nomenclature standards. Data were entered into the WHO Cancer Software CanReg5, and more data quality checks were performed using inbuilt International Agency for Research on Cancer (IARC) Check tools and Person search to detect prevalent cases already registered. Deduplication, secondary cancers, identification of metastasis and consistency checks for all variables entered were also executed. Data were exported from CanReg5 as coma-separated values and converted into Microsoft Excel files. Re-coding of electronic records exported in MS Excel format from the District Health Information System 2 capture module used by health facilities in the Bunyoro sub-region was performed according to the WHO ICD-03 coding theme. Duplicates were also removed to improve the quality of the data using person search functions in CanReg5 Software. Data quality checks for existing records included probabilistic matches for patient names, age and sex. Records of cancer cases from all sub-regions merged into one file and reallocation to the district of origin were done as per the usual residence address and the catchment area of the sub-regions demarcated as satellite cancer registry study sites.

### Population

#### Demographic profile in Uganda

Uganda’s population is projected to be 45.5 million by 2023, and administratively has 146 districts grouped into 15 sub-regions. In this survey, the study used existing administrative units of Uganda, from which 55 districts emerged, forming 10 sub-regions as satellite cancer registry study sites. Five of the sub-regions, namely Buganda, Toro, Sebei, Lango and Elgon, were fused with an ethnically similar sub-region or proximal satellite registry study site [11]. The 10 sub-regions represent all the major ethnic groups in Uganda, catering to any genetic variation as a risk factor for cancer incidence. The Sub-Regions included the Central region, Mid-North, Bugisu, Busoga, Kigezi, Rwenzori, Karamoja, Bukedi, Ankole and West Nile Sub-regions.

**Figure 2.**
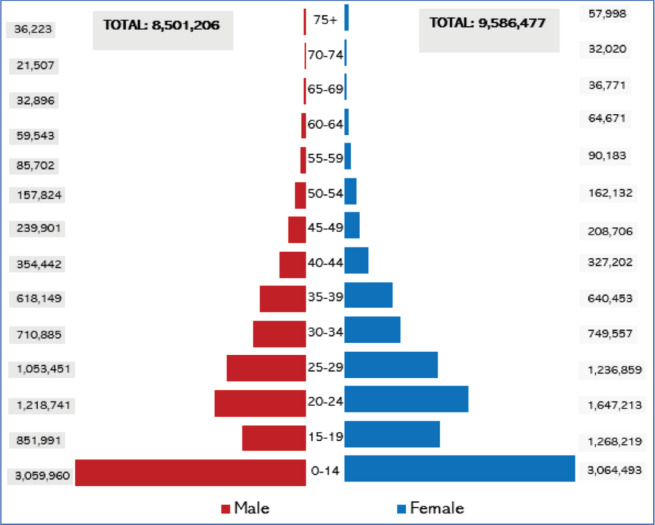
Sub-regional population pyramid (average population 2017–2020).

**Figure 3.**
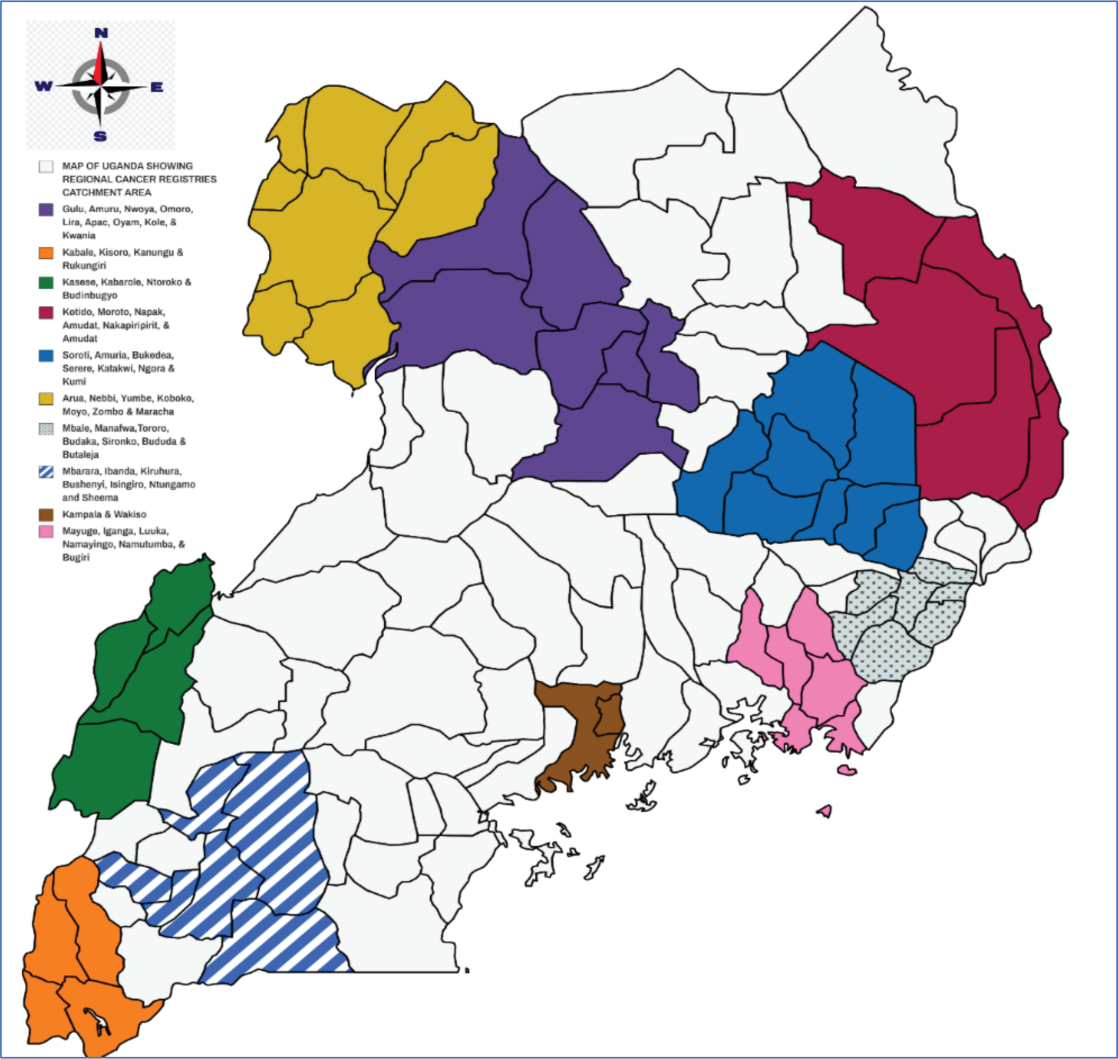
Map of Uganda showing the surveyed sub-regions and their respective districts sampled.

A total catchment population of 18,087,683, representing 43% (Figure 2) of the 42 million Ugandan population, was surveyed for common cancers diagnosed in 2017–2020 compared to the current 5 million (11.9%) catchment by Gulu and Kampala PBCRs. The sub-regions and respective districts surveyed with their total population coverage included the Central Region (3,685,025), mid-north (2,167,950), West Nile (2,098,625), Ankole (2,066,050), Bugishu (2,038,950), Busoga (1,874,525), Teso (1,527,018), Rwenzori (1,236,350), Kigezi (995,650) and Karamoja (397,550). In Uganda, the intercensal population growth rate was 3.0% between 2002 and 2014, while the projected intra censual yearly population growth rate between 2017 and 2020 is constant at 3.1% [4]. The catchment area of the population-based cancer registration survey was well distributed across the sub-regions of Uganda, as shown in Figure 3.

**Figure 4.**
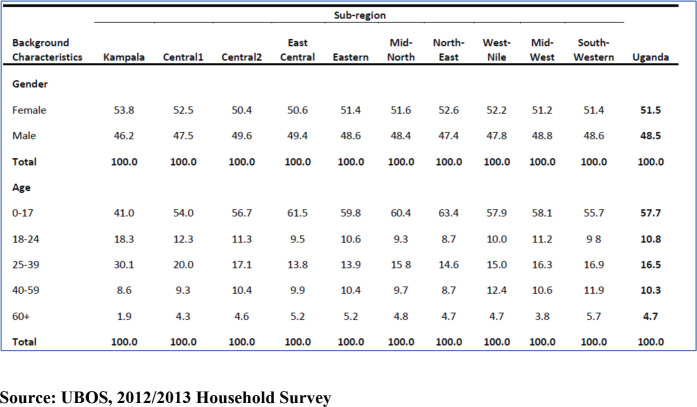
Population characteristics in the sub-regions by sex and age group.

The Uganda Bureau of Statistics (UBOS) findings in the 2012/2013 household survey revealed that females comprise 51.5% of the population and males 48.5% (Figure 4); however, the composition of males and females by each sub-region is consistent across all the regions of Uganda, making sex and age structure across the sub-regions less likely to be a confounding factor in cancer risks by geographical region in Uganda, except for Kampala City, which has a higher proportion of people in the age group 18–39 years corresponding to young men and women migrating for jobs and better education in the city [9].

### Data management and analysis

The World Health Organization CanReg5 software was designed and customized specifically for the study to manage the sub-region cancer data from data entry to coding and cleaning duplicates using person search under the data quality module of CanReg5. Inbuilt IARC check tools were used to confirm the validity of the entered cancer data. The exported comma separated values type of records from CanReg5 were converted to spreadsheet file formats. Electronic cancer records from all other software were re-coded using ICDO-3 and International Classification of Childhood Cancers version 3 (ICCC3) coding themes to match the CanReg5 records. Data from all the sub-regions were merged into one spreadsheet file and patient complete records were posted to each sub-region based on the district of residence forming the satellite registry site regardless of the place of diagnosis. The ‘UNIQUE’ functions in Microsoft Office 365 version spreadsheet were used to return records appearing only once and also remove duplicates from the merged file. Across the field, combination matching for the patient’s name, sex, age, incidence date, address and tumor type was used to check the probabilistic match for any duplicate. The incident date was defined as the date of the initial tumor diagnosis between the first day of January and the last day of December for each period between 2017 and 2020. For subsequent visits, person search data quality features in CanReg5 were used to identify prevalent cases; if the search criterion matched the data entered, the cases were declared duplicate and the case was not re-registered. Any information regarding the patient after diagnosis was updated in the previous records to avoid double registration. Using ICD-10 formats for adult cancers and ICCC3 formats for childhood cancers analysis was done using Microsoft Excel. Univariate descriptive statistics were used to analyze the data using a Microsoft Excel pivot table to estimate cancer incidence crude rates (CRs) by dividing the total number of cases between 2017 and 2020 by the person-years (average population of the catchment area) and multiplying by 100,000. The study also estimated prevalence (total cases between 2017 and 2020) in absolute numbers, and proportions of each tumor by age, sex and sub-regions were also computed.

## Results

### Overall cancer spectrum in Uganda

A total of 25,576 of the 26,881 cancer cases were registered from 55 districts in Uganda for the period 2017–2020 after removing 1,305 duplicate records. Cancer cases were stratified according to the district of usual residence and the sub-regions of Central Uganda, Acholi-Lango, Bugishu, Busoga, Kigezi, Rwenzori, Karamoja, Teso, Ankole and West Nile.

### Cancer CRs in Uganda

The average crude cancer incidence rate in all the sub-regions is 35.4 per 100,000, with the central region having the highest cancer incidence at 63.8 and Karamoja least at 12.8. The top four regions with higher cancer incidences have available cancer diagnostic and treatment services and PBCRs, namely Kampala, Mbarara, Gulu and Mayuge cancer registries, indicating a more complete data collection of the cancer cases diagnosed in their catchment area (Table 1). These CRs are consistent with those in Table 2, where districts such as Kampala, Gulu and Mbarara housing PBCRs also have higher CRs.

Overall, the incidence of cancer in all the sub-regions of Uganda indicates that the top five high cancer burden districts by crude cancer incidence rates are; Kampala at 86.6, Gulu at 73.6, Kabale at 68.1, Iganga at 62.2 and Bushenyi at 56.0 cancer cases for every 100,000 people (Table 3). The districts with the lowest cancer burden by CRs are Sironko at 13.5, Bundibugyo at 13.4, Arua at 12.8, Kanungu at 10.5 and Kole at 10.2 cancer cases per 100,000 inhabitants (Table 4).

Table 3 indicates that the burden of cancer in terms of crude incidences in the most affected districts in Uganda ranges from 86.58 per 100,000 population in Kampala to 73.57 in Gulu, 68.2 in Kabale, 62.22 in Iganga and 54.73 in Mbarara. The rest of the districts had a crude rate of less than 50 cancer cases per 100,000 population. It should be noted that sub-regions such as Karamoja with low health infrastructure for cancer screening and diagnosis could have had low incidence rates because of missed diagnosis of cancers. In the West Nile region, the low crude incidence rates are probably due to inflated person-years at risk due to many refugee settlements and integration in the districts of Yumbe, which was included in the satellite cancer registry study site demarcation.

### Overall sub-regional common cancers by site and sex

In the sub-regions of Uganda, up to 14,322 (56%) cases of cancer were found in females and 11,254 (44%) in males. In Table 5, the top five female cancers included cervical cancer 43% (*n* = 6,190), breast 22% (*n* = 3,200), esophagus 5.6 % (*n* = 800), ovary 5.2% (*n* = 746), Kaposi Sarcoma (KS) 4.7% (*n* = 666) and other less common cancers 18.5 % (2,720).

Table 6 indicates that in males, the top five cancers included prostate cancer 25.1% (*n* = 2,820), esophagus 15.1% (*n* = 1,704), KS 12.4% (*n* = 1,395), liver 8.8% (989) and stomach 4.8% (*n* = 539), and other less common cancers constituted 33.8% (*n* = 3,807). In the sub-regions of Uganda, esophageal cancer is common in both males and females, but the proportion of males is twice that of their female counterparts (Tables 5 and 6).

In children, the top five cancers included lymphomas 33.9% (*n* = 653), soft tissue sarcomas 20.8% (*n* = 400), malignant bone tumors 15.8% (*n* = 305), leukemia other than lymphoid type 13.8% (*n* = 265), myeloid leukemia was more common than lymphoid type and the risk of developing childhood cancer was higher in male child than female, with an average male to female ratio of 1.36:1 (Table 7).

### Cancer spectrum by sub-regions of Uganda

**Figure 5.**
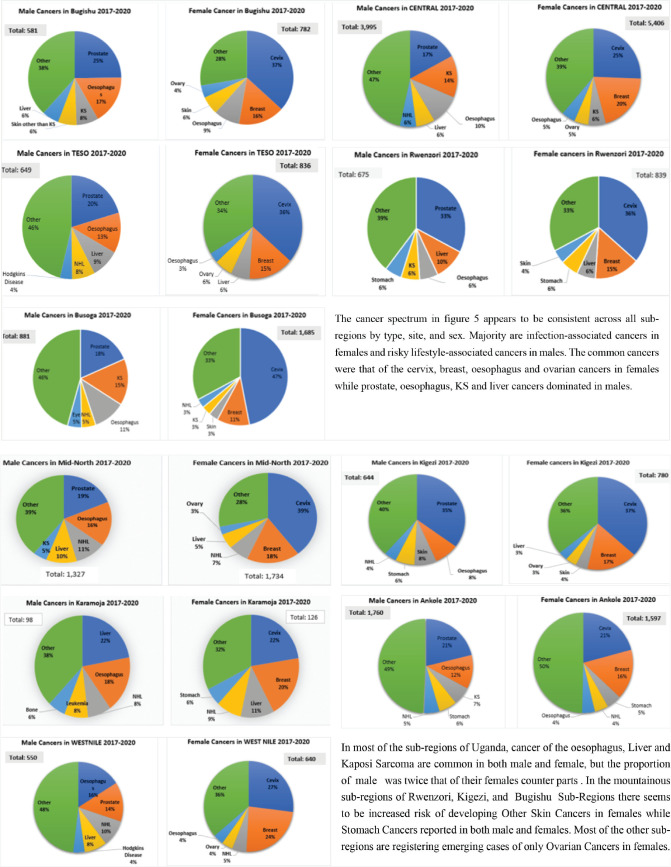
Proportion of cancer spectrum in Uganda by site, sex and sub-regions for the year 2017–2020.

The cancer profile by sex and topography in the ten sub-regions of Uganda was reported for the period 2017–2020. The study highlighted the top five cancers by the number of cases and proportions, as shown in Figure 5.

## Discussion

This population-based survey was conducted to estimate the cancer spectrum in the sub-regions of Uganda, given that the current population-based cancer registration by the Gulu and Kampala Cancer Registries is low. In 2022, the crude cancer incidence rates in Uganda were 74.30 per 100,000, Kenya 79.60 and Tanzania 71.0. The crude incidence rates of male cancers in East Africa range from 57.6 per 100,000 in Tanzania to 58.5 in Kenya and Uganda 64.7, and in females (84.4, 100.3, and 83.4 per 100, 000) [12]. The crude incidence in the sub-regions varies by a wide margin from 63.8 in the Central Region to 12.8 in Karamoja, with an average of 35.4 per 100,000. The variation in crude cancer incidence could be attributed to differences in cancer diagnostics and treatment services and the existence of PBCRs in regions with relatively higher CRs, notwithstanding the minimal effect due to variability in age structure and population growth rates in the sub-regions and between East African countries.

The survey findings indicate that most of the cancer profiles in the sub-regions are similar and close to the Uganda cancer burden reported by the Global Cancer Observatory [13, 14]. The most common cancers in females were those of the cervix (35.7%) and breast (13.7%), consistent with our findings, where 43% and 22% of cases of cervical and breast cancers were registered in all the sub-regions of Uganda, respectively. Ovarian cancers have emerged as one of the most common female cancers in all subregions of Uganda, except for Ankole, Rwenzori, Busoga and Karamoja (Figure 5). It was also surprising to note that ovarian cancers were among the top five cancers in the Central and Mid-Northern regions, where there are PBCRs. This new pattern in the female cancer spectrum could be attributed to the expansion of the cancer registry catchment area during the survey period. There is a growing incidence of ovarian cancer in sub-Saharan Africa [15], which could explain the surfacing of this cancer in all the sub-regions of Uganda. It is not clear whether the risk of mutations in the Breast Cancer Gene 1 (BRCA1) and Breast Cancer Gene 2 (BRCA2) genes can be implicated in the etiology of both breast and ovarian cancers, given the high incidence of female breast cancer globally, in Uganda, or probably due to the improvement in the radiological and immunobiological diagnosis of cancers [16, 17]. Ovarian cancer is one of the emerging female cancers among the previously reported female cancers in the sub-regions of Uganda, and more focus is necessary regarding its screening using radiological means and tumor markers for early detection.

In 2022, the male cancer spectrum by proportion in Uganda includes KS (17.1%) and prostate cancer (16.3%) [2]. This trend is contrary to the results for common cancers in the Ankole sub-region served by the Mbarara population-based cancer registry [18] and our 2017–2020 survey reports where prostate cancer is leading at 25.1%, followed by esophageal cancer at 15.1% and KS at only 12.4%. The difference in overall cancer burden in males highlights the limitations in the coverage of cancer registration in Uganda and the variations in cancer profile and burden by geographical location. In the mid-northern Uganda region, between 2016 and 2021, common cancers in females included Cervical Cancer at 45%, breast cancer at 10.8% and lymphoma at 9.8% [19, 20] tallying with the population survey conducted for cases diagnosed in 2017–2020. In the sub-regions of Uganda, cancers of the esophagus and liver are common in both males and females, but males have a higher proportion than their female counterparts. Esophageal and liver cancers are associated with tobacco use and consumption of harmful alcohol [21], consistent with NCD risk assessment reports in Uganda, which found that consumption of these substances is higher in males than females.

In the Kigezi and Ankole sub-regions, stomach cancer is emerging among the most common cancers in both males and females. In the Mbarara district, located in the Ankole sub-region, cases of stomach cancer are also common compared to all other districts in the region [22]. Other than Helicobacter pylori, risk factors such as diet in the Ankole and Kigezi regions could be associated with the occurrence of this gastrointestinal malignancy in this region.

The cancer spectrum in the sub-regions of Uganda includes the cervix, breast, esophagus, stomach and KS in females. The male counterparts had prostate, liver, esophagus, stomach and KS. Common childhood cancers include lymphomas, sarcomas, bone cancer and myeloid leukemia. Despite similarities in cancer profiles from earlier reports, the survey highlights the emergence of ovarian cancer in females and stomach cancer in both sexes.

Population-level surveillance cancer data that are generalizable to a specific population are essential for assessing the multiple aspects of the cancer control continuum in the given sub-regional population from primary prevention, diagnosis, treatment and survival, and can also enhance cancer surveillance research [23]. In addition, it is estimated that only approximately 0.25% of the sub-Saharan African population is covered by accurate death registration systems that provide data on deaths and the cause of death; thus, the only way to obtain fairly accurate information on cancer burden is through cancer registration [24].

Traditionally, data from PBCR are used to describe cancer burden in a specific community, establish cancer control priorities in specific communities, provide a source of material for cancer etiologic studies and help in planning, monitoring and evaluating national cancer control programs. As such, a national cancer control program should be built on population-based cancer registry data, especially incidence, survival, treatment and treatment outcome data from within its nation or sub-regions [25].

However, recently, the utility of PBCR has expanded beyond the premise of estimating the cancer burden based on incidence, prevalence and mortality to provide much more critical frameworks needed to elevate the science of cancer research in several ways. For example, besides supporting National Cancer Control Plan, PBCR provides population-based sampling frames for cancer control studies with stronger external validity, enhances the completeness of cancer risk data required for health insurance premiums and claims, and through the application of informatics techniques can provide population-based rapid cancer case ascertainment, serve as population-based virtual tissue repositories, guide the implementation of evidence-based interventions and evaluate changes in the cancer burden after the implementation of these interventions [26].

Therefore, these findings emphasize the need for the establishment and support of additional regional PBCRs and periodic population-based cancer surveys to accurately determine the burden of cancer and inform national and sub-national cancer control programs in Uganda. In addition, these data are vital in guiding national cancer control program interventions nationally and by the specific sub-region, given its fair representativeness of all the major sub-regions of Uganda. These data are also crucial in guiding policymakers on priority sub-regions to establish additional regional cancer centers.

Nevertheless, the possibility of limited data completeness and minimal confounding effect of population structure differences across the sub-regions and the study period could not be ruled out, but a pointer to the next step is a nationally representative PBCR system.

## Conclusion

The cancer spectrum survey indicated that in all the sub-regions in Uganda, the top five cancers in women were cancers of the cervix, breast, esophagus, ovary and liver. Prostate and liver cancers dominate the cancer burden in males in Uganda. Esophageal, liver and stomach cancers were the most prevalent in both sexes. In children, Lymphomas, Malignant Bone tumors, soft tissue sarcomas and myeloid leukemias were more common across all regions of Uganda. Most cancer burdensome districts included Kampala at 86.6, Gulu at 73.6, Kabale at 68.1, Iganga at 62.2 and Bushenyi with 56.0 cancer cases for every 100,000 people. Although the cancer profile is similar in most sub-regions of Uganda, except Ankole and subregions with mountainous topography (Rwenzori, Kigezi, Bugisu), there has been significant variation in the cancer profile, especially for males for the entire sub-region compared to those reported for Uganda by PBCRs in Gulu and Kampala. These findings underscore the need for the establishment and support of additional regional PBCRs and periodic population-based cancer surveys to accurately determine the burden of cancer to inform the establishment of regional cancer centers and guide the national and sub-national cancer control programs in Uganda.

### Study limitations

This study acknowledges the limitations of using CRs in estimating cancer incidence, given that in the central region and particularly Kampala City, there is a higher proportion of men and women in the age group 18–39 years compared to other sub-regions. Though the impact of age and sex difference is significantly reduced in all other sub-regions of Uganda where there are similar demographic characteristics, such as age and sex distribution and annual population growth rate, the crude cancer incidence rate is a less reliable measure of incidence in the central region of Uganda compared to the use of age-standardized rate.

## Lists of abbreviations

BRCA1/2, Breast cancer gene 1/2; CR, Crude rates; IARC, International Agency for Research on Cancer; ICCC3, International Classification of Childhood Cancers Version 3; ICD-10, International Classification of Diseases Version 10; ICD-O3, International Classification of Diseases for Oncology Version 3; KS, Kaposi Sarcoma; NCD, Non-communicable diseases; PBCR, Population based cancer registry; UBOS, Uganda Bureau of Statistics; UCI, Uganda Cancer Institute.

## Authors’ contributions

Francis Okongo (Uganda Cancer Institute, Kampala) provided guidance on the content of the data collection tools, conducted the study, analyzed the data and participated in manuscript writing. Jackson Orem (Uganda Cancer Institute) conceptualized, supervised the study and participated in manuscript writing. Nixon Niyonzima (Uganda Cancer Institute), supervised the study and provided guidance on the content of data collection tools and writing of this manuscript. Alfred Jatho (Uganda Cancer Institute), conducted the study and participated in the project reporting and manuscript writing. Catherine Amuge (Uganda Cancer Institute) conducted the study and participated in the project coordination, and manuscript writing. David Martin Ogwang (St. Mary’s Hospital, Lacor) conducted the study, analyzed the data and participated in writing the manuscript.

## Conflict of interest

The authors declare no competing interests.

## Data availability

All relevant data are within the paper.

## Figures and Tables

**Table 1.** Crude incidences by sub-regions of Uganda 2017–2020.

SUB-REGIONS	CANCER CASES	PERSON YEARS AT RISK	CR/10 0,000
CENTRAL	9,401	14,740,000	63.8
WESTERN-ANKOLE	3,357	8,264,200	40.6
MID-NORTH-ACHOLI/LANGO	3,061	8,671,800	35.3
EASTERN-BUSOGA	2,567	7,498,100	34.2
WESTERN-RWENZORI	1,504	4,945,400	30.4
TESO	1,485	6,108,072	24.3
WESTERN-KIGEZI	1,444	3,982,600	36.3
EASTERN-BUGISU	1,363	8,155,800	16.7
WESTNILE	1,190	8,394,500	14.2
KARAMOJA	204	1,590,200	12.8
**TOTAL**	**25,576**	**72,350,672**	**35.4**

**Table 2.** Sub-regional cancer burden and most affected districts.

Sub-regional cancer burden and districts affected		Cancer crude incidence rates per 100,000
1. Mid-north	Gulu	73.57
	Amuru	42.50
2. Kigezi	Kabale	68.12
	Kisoro	30.41
3. Karamoja	Nakapiripirit	23.71
	Kotido	7.01
4. Rwenzori	Kabarole	45.78
	Kasese	20.82
5. West Nile	Nebbi	21.32
	Moyo	17.26
6. Central	Kampala	86.58
	Wakiso	35.02
7. Teso	Soroti	46.51
	Kumi	30.45
8. Busoga	Iganga	62.22
	Mayuge	31.77
9. Bugishu	Manafwa	18.29
	Mbale	14.57
10. Ankole	Bushenyi	55.97
	Mbarara	54.73

**Table 3.** Top five high burden districts by cancer cases and CRs for the period 2017–2020.

Districts	Cancer Cases	Person Years	CR/100,000
**KAMPALA DISTRICT**	**5,664**	** 6,542,100**	**86.6**
**GULU DISTRICT**	**919**	** 1,249,100**	**73.6**
**KABALE DISTRICT**	**665**	** 976,200**	**68.1**
**IGANGA DISTRICT**	**960**	** 1,542,800**	**62.2**
**BUSHENYI DISTRICT**	**548**	** 979,100**	**56.0**
MBARARA DISTRICT	1,136	2,075,500	54.7
SOROTI DISTRICT	643	1,382,400	46.5
KABAROLE DISTRICT	600	1,310,700	45.8
AMURU DISTRICT	359	835,200	43.0
Omoro District	274	714,100	38.4
WAKISO DISTRICT	3,737	10,671,100	35.0
APAC DISTRICT	276	861,800	32.0
MAYUGE DISTRICT	687	2,162,100	31.8
KUMI DISTRICT	332	1,090,400	30.4
KISORO DISTRICT	373	1,226,500	30.4
BUGIRI DISTRICT	534	1,815,500	29.4
IBANDA DISTRICT	288	1,080,400	26.7
OYAM DISTRICT	460	1,740,300	26.4
LIRA DISTRICT	464	1,839,100	25.2

**Table 4.** Five least burden districts by cancer cases and CRs for the period 2017–2020.

District	Cancer Cases	Person Years	CR/100,000
LIRA DISTRICT	464	1,839,100	25.2
NWOYA DISTRICT	198	820,700	24.1
NAKAPIRIPIRIT DISTRICT	101	425,900	23.7
RUKUNGIRI DISTRICT	296	1,316,000	22.5
NAMAYINGO District	205	925,700	22.1
KIRUHURA DISTRICT	338	1,532,000	22.1
NEBBI DISTRICT	293	1,374,100	21.3
Sheema District	182	869,800	20.9
KATAKWI DISTRICT	156	748,100	20.9
KASESE DISTRICT	770	3,698,000	20.8
NTUNGAMO DISTRICT	435	2,104,100	20.7
AMURIA DISTRICT	175	855,100	20.5
MANAFWA DISTRICT	124	677,900	18.3
MOYO DISTRICT	106	614,000	17.3
LUUKA District	178	1,038,100	17.1
ISINGIRO DISTRICT	430	2,866,800	15.0
MBALE DISTRICT	475	3,260,100	14.6
TORORO DISTRICT	332	2,305,200	14.4
BUKEDEA DISTRICT	140	976,300	14.3
MARACHA DISTRICT	112	810,300	13.8
BUDAKA DISTRICT	133	963,300	13.8
**SIRONKO DISTRICT**	**144**	** 1,063,900**	**13.5**
**BUNDIBUGYO DISTRICT**	**134**	** 998,000**	**13.4**
**ARUA DISTRICT**	**449**	** 3,519,800**	**12.8**
**KANUNGU DISTRICT**	**110**	** 1,048,200**	**10.5**
**KOLE DISTRICT**	**111**	** 1,089,300**	**10.2**

**Table 5.** Showing common female cancers in all the sub-regions of Uganda (2017–2020).

CANCERS BY SITE-FEMALE	ICD-10	CANCER CASES	PERCENT
Cervix uteri	C53	6,190	43
Breast	C50	3,200	22
Oesophagus	C15	800	5.6
Ovary	C56	746	5.2
Kaposi sarcoma	C46	666	4.7
Others		2,720	18.5
**TOTAL**		**14,322**	**100**

**Table 6.** Showing common male cancers in all the sub-regions of Uganda (2017–2020).

CANCERS BY SITE-MALE	ICD-10	CANCER CASES	PERCENT
Prostate	C61	2820	25.1
Oesophagus	C15	1,704	15.1
Kaposi Sarcoma	C46	1395	12.4
Liver	C22	989	8.8
Stomach	C16	539	4.8
Others		3807	33.8
**TOTAL**		**11,254**	**100**

**Table 7.** Showing common childhood cancers in all the sub-regions of Uganda (2017–2020).

CHILDHOOD CANCERS U15 YEARS BY ICCC3 CATEGORY	ICCC CODE	CASES	M/F Ratio	% Total
Lymphomas NOS	**2a-e**	653	1.6	**33.9**
Soft Tissue Sarcomas	**9a-d**	400	1.2	**20.8**
Malignant Bone Tumours	**8a-e**	305	1.2	**15.8**
Leukaemias other than lymphoid type	**1a-e**	265	1.2	**13.8**
**Others**		**303**	**1.6**	**15.7**
**TOTAL**		**1,926**	**1.36**	**100**
